# High prevalence of *Eucoleus boehmi* (syn. *Capillaria boehmi*) in foxes from western Austria

**DOI:** 10.1007/s00436-016-5145-8

**Published:** 2016-05-27

**Authors:** Adnan Hodžić, Pia Bruckschwaiger, Georg Gerhard Duscher, Walter Glawischnig, Hans-Peter Fuehrer

**Affiliations:** Institute of Parasitology, Department of Pathobiology, University of Veterinary Medicine Vienna, Veterinaerplatz 1, 1210 Vienna, Austria; Institute for Veterinary Disease Control, Austrian Agency for Health and Food Safety, Technikerstraße 70, 6020 Innsbruck, Austria

**Keywords:** *Eucoleus boehmi*, Red fox, *Vulpes vulpes*, Tyrol, Vorarlberg, Austria

## Abstract

*Eucoleus boehmi* (syn. *Capillaria boehmi*) is a canine trichuroid nematode affecting the upper respiratory airways (i.e., nasal cavity and paranasal sinuses) of dogs, foxes, and wolves. In the past few years, reports in dogs and wild canids have increased from across Europe, but data on its occurrence and distribution in Austria is scanty. A total of 47 red foxes (*Vulpes vulpes*) from the two westernmost provinces (Tyrol and Vorarlberg) of Austria were therefore examined for the presence of *E. boehmi* at necropsy. Eggs and adult nematodes were identified morphologically and molecularly (*cox*1) as *E. boehmi*. These nematodes were found in 26 (78.8 %) and 13 (92.9 %) foxes from Tyrol and Vorarlberg, respectively, with an overall prevalence of 83.0 % (39/47). The prevalence rate of infection recorded in this study is among the highest in Europe. These results suggest that foxes may represent an important source of infection for dogs and other canids, but further studies are needed to elucidate the transmission dynamics.

## Introduction

*Eucoleus boehmi* (syn. *Capillaria boehmi*) is a poorly studied capillarid nematode that inhabits the mucosa of the nasal cavity and paranasal sinuses of domestic dogs, foxes, and wolves (Veronesi et al. 2014; Di Cesare et al. 2015). This nematode was described for the first time in red foxes from Lower Austria in 1953, although the characteristic eggs of the parasite were found in fecal samples of silver foxes originating from Moravia, Czech Republic, many years before (1929) by Professor Leopold Karl Boehm, after whom the species is named (Supperer 1953). The biological life cycle and route(s) of transmission of this nematode are still unknown, but it is hypothesized that animals become infected by ingestion of eggs containing the infective larvae or by ingestion of earthworms which may act as facultative intermediate or paratenic host (Traversa et al. 2010; Veronesi et al. 2013; Di Cesare et al. 2015). In dogs, infections with *E. boehmi* often remain asymptomatic, but in case of higher parasite burden, animals may show distress of the upper respiratory tract with varying clinical signs, such as cough, sneezing, reverse sneezing, wheezing, epistaxis, nasal discharge, and hypo- or anosmia (Traversa et al. 2010; Veronesi et al. 2013, 2014; Di Cesare et al. 2015; Morganti et al. 2015; Alho et al. 2016). Moreover, infection with this parasite has recently been recognized as a potential cause of chronic meningoencephalitis and consequently convulsive seizures in dogs (Clark et al. 2013).

Red foxes (*Vulpes vulpes*) are assumed to be the main reservoir host of *E. boehmi* and assumed to play a major role in rising numbers of cases of infection in domestic dogs worldwide (Traversa et al. 2010; Veronesi et al. 2014). The growing population of foxes along with their rapid urbanization increases the risk of infections in pets and spreading of the parasites to non-endemic areas (Saeed et al. 2006; Traversa et al. 2010; Veronesi et al. 2014; Otranto et al. 2015). However, despite the pathogenic potential and apparent emergence of *E. boehmi* in several European countries, it is still a neglected and underestimated cause of respiratory diseases (Veronesi et al. 2013, 2014; Di Cesare et al. 2015). Data on the occurrence and geographical distribution of *E. boehmi* in Austria are fragmentary and obsolete; therefore, the aim of the present study was to investigate the occurrence of this nematode species in foxes from western Austria.

## Materials and methods

Carcasses of 47 red foxes originating from two provinces in western Austria (Tyrol and Vorarlberg) were collected by hunters and delivered to the Institute for Veterinary Disease Control in Innsbruck, Austria, as a part of a *Trichinella* control program. All animals were shot legally under the restrictions of the Austrian game laws between December 2014 and August 2015. Data on geographical location, sex, and age were documented for each animal individually. The animals were classified as juveniles (<1 year) or adults (>1 year) based on dentition and levels of tooth wear. At necropsy, the heads of all foxes were removed and kept frozen at −80 °C until sent to the Institute of Parasitology, University of Veterinary Medicine Vienna for further parasitological examination. In order to investigate the occurrence of *E. boehmi*, the heads were longitudinally cut into two halves by using an oscillating saw and the nasal cavity and sinuses were examined for adult worms. In addition, nasal mucosa was scraped and flushed into a conical glass (300 ml) with water. After 30 min sedimentation at room temperature, supernatant and small pieces of the tissue were discarded. The sediment was then transferred to a Petri dish and inspected under a stereomicroscope (×10–40 magnification) for the presence of additional worms and/or eggs (Veronesi et al. 2014). Animals were considered positive if adult worms and/or eggs were found. Nematodes and eggs recovered were identified to species level based on previous descriptions (Supperer 1953; Moravec 2000; Traversa et al. 2010; Di Cesare et al. 2012a; Lalošević et al. 2013).

Molecular characterization and identity confirmation of specimens collected were performed by conventional PCR (Di Cesare et al. 2012b). Briefly, genomic DNA was extracted from adult worms (*n* = 4) previously stored in 70 % ethanol using the High Pure PCR Template Preparation Kit (Roche Diagnostics, Germany) in accordance with the manufacturer’s instructions. A 344-bp long fragment of the mitochondrial *cox*1 gene was amplified using the set of primers Cox1NEMF (5′CCTGAGGTTTATATTYTWRTT-3′) and Cox1NEMR (5′CCTGTTARRCCTCCRATACT-3′) specific for the Capillarinae subfamily (Di Cesare et al. 2012b). PCR was carried out in a final volume of 25 μl using 5× Green Reaction Buffer and GoTaq G2® Polymerase (Promega, Germany). PCR products were visualized by electrophoresis on 2 % agarose gels stained with Midori-Green Advance® (Biozym, Germany). The amplicons were purified and sequenced in both directions by a commercial company (Microsynth, Austria). Sequences obtained were aligned and compared with those available in the GenBank® database using Basic Local Alignment Search Tool (BLAST) analysis.

Statistical analysis was performed using SPSS 20.0 statistical software. The chi-square test was used to compare the differences in prevalence rates among the region, sex, and age of the animals. Differences were considered significant if *p* value was lower than 0.05.

## Results and discussion

Out of 47 foxes collected from Tyrol (*n* = 33) and Vorarlberg (*n* = 14), 16 were males and 31 were females; 29 individuals were classified as juveniles and 18 as adults. Eggs and adult nematodes were found in 26 (78.8 %) and 13 (92.9 %) foxes from Tyrol and Vorarlberg, respectively, corresponding to an overall prevalence of 83.0 % (39/47) (Table 1). All animals tested positive at the visual inspection of the nasal cavity were also positive with the scraping and flushing technique. Worms and/or eggs of the parasite could not be found in paranasal sinuses in any of the tested animals. No significant differences in the prevalence rate between geographical region (*p* = 0.451), sex (*p* = 0.518), and age (*p* = 0.581) of the host were recorded. In total, 249 adult worms were recovered from the nasal cavity of the infected animals. The intensity of infection ranged from two to 20 nematodes per animal, with a mean abundance of 6.4 (Table 1).

**Table 1:** Prevalence and intensity of *Eucoleus boehmi* infection in foxes from western Austria

Origin	Host data							Nematode data	
	Male	Female	<1 year	>1 year	Total	%	95 % CI	Number	Range (mean ± SD)
Tyrol	7	19	14	12	26	78.8	62.2–89.3	158	2–18 (6.0 ± 4.2)
Vorarlberg	7	6	10	3	13	92.9	68.5–98.7	91	2–20 (7.0 ± 4.5)
Total	14	25	24	15	39	83.0	69.9–91.1	249	2–20 (6.4 ± 4.3)

*CI* confidence interval, *SD* standard deviation

All nematode specimens collected were morphologically identified as *E. boehmi*. The identification was further confirmed by PCR and sequencing. The *cox*1 sequences displayed 99–100 % identity with the nucleotide sequence of *E. boehmi* (GenBank® accession no. KR186213), previously found in dogs with clinical signs of nasal eucoleosis in Italy (Di Cesare et al. 2015). Sequences obtained in this study were deposited in GenBank® database (accession nos. KX027311-KX027314).

The first comprehensive study on endo- and ectoparasites in populations of red foxes in Austria was conducted in 1971, and 14 (14.0 %) out of a total of 100 animals collected from six provinces (Styria, Lower Austria, Upper Austria, Carinthia, Salzburg, and Burgenland) were found to be positive for *E. boehmi* (Hinaidy 1971). Previously reported prevalences in foxes varied from 8 % in Hungary (Sréter et al. 2003) and 30.7 % in Germany (Ballek et al. 1992; Janka and Stoye 1998) to 50 % in Italy (Veronesi et al. 2014; Magi et al. 2015) and 51 % in Norway (Davidson et al. 2006). The highest prevalence (90 %) has been recorded in foxes in Serbia, but only ten animals were examined (Lalošević et al. 2013). The mean intensity of worm burden observed in our study is higher than those reported in foxes from Hungary (Sréter et al. 2003) and Italy (Veronesi et al. 2014). The differences in the prevalence rates and the intensity of infections may be due to the different recovery techniques used, variations in environmental factors (i.e., temperature, humidity), abundance of intermediate host(s), and size of fox populations (Veronesi et al. 2014).

**Fig. 1:**
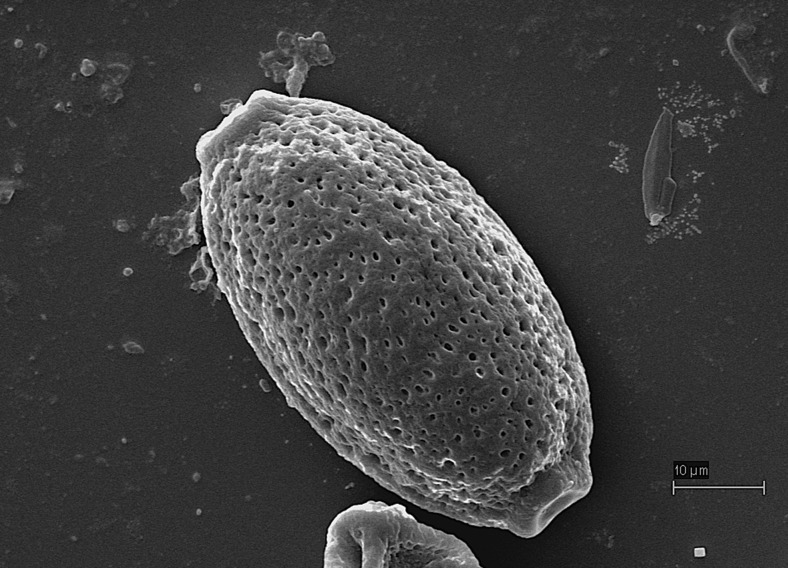
Scanning electron microscopy (SEM): egg of *Eucoleus boehmi* showing the characteristic pitted wall surface. Courtesy of Professor Salvatore Giannetto, University of Messina, Italy. *Scale bar* = 10 μm

Despite the similar localization of adult worms of *E. boehmi* (i.e., nasal meatuses, caudal ethmoidal turbinate) and pathological changes with varying severity in dogs, its pathogenic potential and clinical impact on wild canids are still unknown (Veronesi et al. 2013, 2014). In dogs, nasal eucoleosis is likely to be underestimated mostly because of the lack of specificity of clinical signs and difficulties in morphological identification of eggs (Traversa et al. 2010; Di Cesare et al. 2012a; Morganti et al. 2015). High morphological similarity of *E. boehmi* eggs to those of *Eucoleus aerophilus* (syn. *Capillaria aerophila*) and *Trichuris vulpis* (that also can occur in companion animals) may lead to misdiagnosis, especially in mixed infections (Traversa et al. 2010; Di Cesare et al. 2012b, 2015). Eggs of *E. boehmi* are 50–60 μm long and 30–35 μm wide, containing multicellular embryos that do not fill the egg and have a delicately pitted surface (Fig. 1). The eggs of *E. aerophilus* (60–65 × 25–40 μm) and *T. vulpis* (72–94 × 31–42 μm) are bigger in size and have a net-like ornamented outer shell and a thick and completely smooth surface, respectively (Traversa et al. 2011; Di Cesare et al. 2012a). DNA-based assays for the molecular specification of *E. aerophilus* and *E. boehmi* have therefore recently been suggested to avoid misdiagnosis (Di Cesare et al. 2012b, 2015; Guardone et al. 2013).

In conclusion, the present study indicates a high prevalence of *E. boehmi* in foxes in western Austria and suggests that they may have a relevant impact in the transmission of this parasite to domestic dogs. Therefore, veterinarians should be aware of its occurrence and include nasal eucoleosis in the differential diagnosis of upper respiratory diseases in dogs. Moreover, further molecular studies including domestic and wild canids from different geographical regions are needed to elucidate the transmission dynamics of *E. boehmi* and to clarify its role in causing respiratory diseases in wild canids.
